# LINC01002 functions as a ceRNA to regulate FRMD8 by sponging miR-4324 for the development of COVID-19

**DOI:** 10.1186/s12985-024-02382-2

**Published:** 2024-05-11

**Authors:** Xinyi Kong, Qinjin Wang, Xumeng Wang, Kaming Yang, Shuping Nie, Yuetong Li, Wanwen Lao, Xin Yu, Yanping Zhang, Zhenlin Li, Yang Liu, Jie Ning, Yan Wang, Changlong Bi, Chao Wu, Aixia Zhai

**Affiliations:** 1 Department of Laboratory Medicine, The Eighth Affiliated Hospital, Sun Yat-sen University, Shenzhen, 518033 China; 2 Department of Endocrinology, The Eighth Affiliated Hospital, Sun Yat-sen University, Shenzhen, 518033 China; 3 Department of Microbiology, Harbin Medical University, Harbin, 150081 China

**Keywords:** COVID-19, SARS-CoV-2, Interferon, FRMD8, LINC01002, miR-4324

## Abstract

**Background:**

Syndrome coronavirus-2 (SARS-CoV-2) has developed various strategies to evade the antiviral impact of type I IFN. Non-structural proteins and auxiliary proteins have been extensively researched on their role in immune escape. Nevertheless, the detailed mechanisms of structural protein-induced immune evasion have not been well elucidated.

**Methods:**

Human alveolar basal epithelial carcinoma cell line (A549) was stimulated with polyinosinic-polycytidylic acid (PIC) and independently transfected with four structural proteins expression plasmids, including nucleocapsid (N), spike (S), membrane (M) and envelope (E) proteins. By RT-qPCR and ELISA, the structural protein with the most pronounced inhibitory effects on IFN-β induction was screened. RNA-sequencing (RNA-Seq) and two differential analysis strategies were used to obtain differentially expressed genes associated with N protein inhibition of IFN-β induction. Based on DIANA-LncBase and StarBase databases, the interactive competitive endogenous RNA (ceRNA) network for N protein-associated genes was constructed. By combining single-cell sequencing data (GSE158055), lncRNA-miRNA-mRNA axis was further determined. Finally, RT-qPCR was utilized to illustrate the regulatory functions among components of the ceRNA axis.

**Results:**

SARS-CoV-2 N protein inhibited IFN-β induction in human alveolar epithelial cells most significantly compared with other structural proteins. RNA-Seq data analysis revealed genes related to N protein inhibiting IFNs induction. The obtained 858 differentially expressed genes formed the reliable ceRNA network. The function of LINC01002-miR-4324-FRMD8 axis in the IFN-dominated immune evasion was further demonstrated through integrating single-cell sequencing data. Moreover, we validated that N protein could reverse the effect of PIC on LINC01002, FRMD8 and miR-4324 expression, and subsequently on IFN-β expression level. And LINC01002 could regulate the production of FRMD8 by inhibiting miR-4324.

**Conclusion:**

SARS-CoV-2 N protein suppressed the induction of IFN-β by regulating LINC01002 which was as a ceRNA, sponging miR-4324 and participating in the regulation of FRMD8 mRNA. Our discovery provides new insights into early intervention therapy and drug development on SARS-CoV-2 infection.

**Supplementary Information:**

The online version contains supplementary material available at 10.1186/s12985-024-02382-2.

## Introduction

The coronavirus disease 2019 (COVID-19) pandemic triggered by severe acute respiratory syndrome coronavirus-2 (SARS-CoV-2) has harmed billions of people globally and devastated the economy. It is thought to have emerged from a zoonotic source and spread quickly among humans through respiratory droplets and close contact [1]. This global health crisis has had a severe negative impact on the lives of patients and their families. SARS-CoV-2, like SARS-CoV-1 and Middle East respiratory syndrome coronavirus (MERS-CoV), is a member of the genus *Betacoronavirus* within the family *Coronavirida* [2]. The genome-wide sequencing of SARS-CoV-2 has 79% identity with SARS-CoV-1 and 50% identity with MERS-CoV [1]. As with SARS and MERS, COVID-19 may be life-threatening to patients. Patients with COVID-19 infection usually develop pneumonia-associated symptoms first, followed by life-threatening complications [3], such as pulmonary failure, acute respiratory distress syndrome (ARDS), thromboinflammation, multiple organ dysfunction syndrome (MODS), and death [4–6]. According to the Center for Systems Science and Engineering (CSSE) at Johns Hopkins University, as of March 10, 2023, SARS-CoV-2 has infected 676,609,955 people and caused 6,881,955 deaths since the first outbreak in December 2019 (https://coronavirus.jhu.edu/map.html).

SARS-CoV-2 is an enveloped virus of 30 kb, consisting of a positive-sense, single-stranded RNA genome. For viruses to grow and reproduce, they use the viral genome as a guide to create positive-sense genomic RNA (gRNA) and subgenomic RNAs (sgRNA). The gRNA is subsequently packed into structural proteins (spike, membrane, and envelope) to finish the progeny viral assembly [7]. Especially, the infection caused by SARS-CoV-2 triggers both the innate and adaptive immune responses, which play a crucial role in resolving COVID-19. A fast and well-coordinated immune response is the first line of defense against viral infection. Insufficient protective immune responses and excessive inflammatory reactions may lead to harmful tissue damage at the virus entry site and systemic level, suggesting that the host immune system may be affected throughout COVID-19 development [5, 8]. Interferons (IFNs) are central to antiviral immunity [9]. The absence of IFNs production or sensing can make individuals more vulnerable to a wide range of viral infections [10]. Type I IFN, including IFN-α and IFN-β, is an innate cytokine critical for the initial defense against viruses. Intranasal treatment with type I IFN during early SARS-CoV-2 infection resulted in reduced viral replication and inflammation and decreased transmission [11]. Similar to other viruses, SARS-CoV-2 has developed various strategies to evade the antiviral impact of type I IFN and dysregulated IFNs responses are associated with the immunopathogenesis of viral infection [12]. Intriguingly, a distinct phenotype was observed in severe and critical COVID-19 patients, consisting of a significantly compromised type I IFN response characterized by no IFN-β and low IFN-α production and activity [9]. By comparison, in nonhospitalized patients with mild flu symptoms, type I IFN was markedly induced earlier and at higher levels [13]. These data suggest that type I IFN induction deficiency could be a hallmark of severe COVID-19. Remarkably, quite a few SARS-CoV-1 proteins, including structural proteins and accessory proteins, have inhibitory effects on type I IFN-mediated antiviral immune responses [12, 14, 15]. Due to the high consistency of gene sequence, many SARS-CoV-2 proteins are expected to have inhibitory effects on type I IFN responses similar to those of SARS-CoV-1 proteins.

Existing studies have indicated that the genome of SARS-CoV-2 encodes nucleocapsid (N), spike (S), membrane (M), envelope (E) proteins, accessory proteins including open reading frame (ORF) 3a, 3b, 6, 7a, 7b, 8, 9b, 9c and 10, and two main ORFs of SARS-CoV-2, ORF1a and ORF1b, which encode 16 nonstructural proteins (NSPs) and account for over 70% of the viral genome [16, 17]. To date, the NSPs and accessory proteins of SARS-CoV-2, including NSP1, NSP3, NSP5, NSP6, NSP8, NSP12, NSP13, NSP14, NSP15, ORF3a, ORF6, ORF7b, ORF8, ORF9b, ORF10 and so on, have been well studied [18–21]. Researches showed that those proteins play indispensable roles in suppressing the production and signaling of type I IFNs. As for structural proteins, M protein has been proven to impair mitochondrial antiviral signaling proteins (MAVS) aggregation and its recruitment of downstream components such as TNF receptor-associated factor 3 (TRAF3), TANK-binding kinase 1 (TBK1), and mitochondrial antiviral signaling proteins (IRF3), inhibiting IFN-β induction [18, 22]. However, E protein and S protein exert opposite effects. E protein could be recognized by TLR2, leading to inducing the release of inflammatory cytokines, including TNF-α and IFN-γ, which indicates an excessive inflammatory response or cytokine storm [23]. And S protein induced type I IFN responses via cell fusion and cGAS-STING pathway [24]. As the most abundant viral protein during SARS-CoV-2 infection, N protein is considered one of the critical viral innate antagonists. It’s found that the infection of viral strains with increased N protein expression may be related to lower expression and secretion of IFN-β and N protein could repress retinoic acid-inducible gene (RIG)-mediated IFN-β production [25, 26]. A study also demonstrates that SARS-CoV-2 N protein dually regulates innate immune responses. The low-dose N protein suppressed type I IFN signaling and inflammatory cytokines, whereas the high-dose N protein promoted type I IFN signaling and inflammatory cytokines [27]. Nevertheless, the detailed mechanisms of immune evasion inducted by structural proteins are not well elucidated. SARS-CoV-2 infection was characterized by an absence of circulating IFN-β in COVID-19 patients with all disease-severity grades [9]. In this study, we investigated the impact of four structural proteins on the expression of IFN-β and identified the SARS-CoV-2 N protein as a key mediator in inhibiting IFN-β induction. The molecular mechanism of N protein leading to IFN-β altered expression remains to be investigated during SARS-CoV-2 infection of the organism. This may be a key point in overcoming SARS-CoV-2 immune escape.

Here, we investigate the relationship between SARS-CoV-2 N protein and IFN-β expression. Then we demonstrated that N protein suppressed the induction of IFN-β by regulating LINC01002 which was as a ceRNA, sponging miR-4324 and participating in the regulation of FERM domain-containing protein 8 (FRMD8) mRNA. Targeting the N protein-dependent LINC01002-miR-4324-FRMD8 axis may provide a novel therapeutic idea for early intervention in the IFN-dominated immune evasion induced by SARS-CoV-2, thereby ameliorating the severe inflammatory response caused by COVID-19. Our results provide mechanistic insights into the relationship between SARS-CoV-2 N protein and IFN-β induction suppression.

## Materials and methods

### Cell culture and transfection

Human alveolar basal epithelial carcinoma cell line (A549) and non-small cell lung cancer cell line (H460) were placed in Dulbecco’s Modified Eagle Medium (DMEM) containing glucose (4.5 g/L) (Gibco, C11995500BT) supplemented with 10% fetal bovine serum (FBS) (BioInd, 04-001-1ACS-1) and 1% (v: v) penicillin-streptomycin (Gibco, 15,140,122). The culture environment of 37 °C and 5% CO2 concentration was also maintained. The gene sequences (NCBI Reference Sequence: NC_045512.2) of SARS-CoV-2 M, S, E, and N protein were synthesized and respectively cloned into an expression vector (pEGFP-N1). The construction of the expression plasmids was completed by MiaoLing Plasmid Platform (Wuhan, China). All vectors were verified by sequencing. The siRNAs of LINC01002 and FRMD8 have been supplemented to design and synthesize by IGE Biotechnology (Guangzhou, China), and the mimics and inhibitors of miR-4323 have been supplemented to design and synthesize by GenePharma (Shanghai, China). Specific sequences can be found in Table S1. The plasmids, siRNAs, miRNA mimics and inhibitors were transfected into A549 cells and H460 cells using lipofectamine 2000 transfection reagent (Invitrogen, 11,668,019) according to the manufacturer’s protocol.

### Quantitative reverse transcription-PCR (RT-qPCR)

A549 cells and H460 cells were transfected with the plasmids of SARS-CoV-2 structural protein for 24 h, then were treated with 10 µg/mL polyinosinic-polycytidylic acid (PIC) (Sigma-Aldrich, USA) to incubate for 4 h. PIC acts as a dsRNA mimic to activate immune cells and induce the production of Type I IFN and other immune-related molecules [28]. TRIzol (Invitrogen, 15,596,018) was used to extract total RNA from the cells. Total RNA was reverse transcribed with PrimeScript™ RT Master Mix (TaKaRa, #RR036A), and complementary DNA was analysed by qPCR using TB Green® Premix Ex Taq™ II (TaKaRa, #RR820A). The relative expression was determined using the 2^−ΔΔCT^ method and transcript levels were normalised to the levels of *GAPDH* mRNA expression. In addition, reverse transcription of miR-4324 was performed by utilizing the miRNA 1st strand cDNA synthesis kit (Stem-loop) (Accurate Biology, #AG11743). QPCR was performed with the same kit as above and the relative expression of miRNAs was normalized to U6. All primer sequences are listed in Table S2.

### ELISA

The plasmids of SARS-CoV-2 structural protein were transfected cells for 24 h, then were treated with PIC to incubate for 24 h and cell culture supernatant was harvested. IFN-β level was measured using Human Interferon Beta ELISA Kit (MEIKE, China) according to the manufacturer’s instructions.

### Western blotting (WB)

Cells were lysed in RIPA Buffer (Thermo, USA) supplemented with PMSF Protease Inhibitor (Thermo, USA). Protein concentrations were measured and adjusted accordingly using the BCA Protein Quantification Kit (Beyotime, China). Equal amounts of protein extract were loaded onto gels for SDS-PAGE. The proteins were then transferred to a PVDF Membrane (Millipore, USA) and blocked with 5% non-fat milk in a shaker for 1.5 h at 37 °C. The membrane was incubated with indicated primary antibodies at 4 °C overnight, including anti-SARS-CoV-2 N (Proteintech Group, #28769-1-AP, 1:1000), anti-SARS-CoV-2 S (Proteintech Group, #67758-1-Ig, 1:5000), anti-SARS-CoV-2 M (Cell Signaling Technology, #32,904, 1:1000), anti-SARS-CoV-2 E (Cell Signaling Technology, #74,698, 1:1000), and anti-GAPDH (ZSGB-BIO, #TA-08, 1:1000). Subsequently, secondary antibody against rabbit (ZSGB-BIO, #ZB-2301, 1:1000) or mouse (ZSGB-BIO, #ZB-2305, 1:1000) was incubated for 1 h at room temperature. After washing with TBST, the membrane was visualized by ECL Chemiluminescent Substrate (Thermo, USA). Data was obtained and calculated using Image Lab and ImageJ software.

### RNA-sequencing (RNA-Seq)

A549 cells were divided into four treatment groups: the first and second groups were transfected with pEGFP-N1 plasmid (NC), the third and fourth groups were transfected with SARS-CoV-2 N plasmid. 48 h later, the second and fourth groups were transfected with PIC for 4 h. Samples were collected and sent to transcriptome sequencing.

After completion of RNA quantification and qualification, we purified mRNA from total RNA using poly-T oligo-attached magnetic beads and completed cDNA synthesis. The library fragments were then purified using the AMPure XP system (Beckman Coulter, USA) to obtain cDNA fragments. Subsequently, PCR reactions were performed using Phusion high-fidelity DNA polymerase, universal PCR primers, and index (X) primers, and the AMPure XP system was used to purify the PCR products. After assessing the quality of the library, we used the TruSeq PE Cluster Kit v3-cBot-HS (Illumia) to cluster index-coded samples on the cBot Cluster Generation System and sequenced them on the Illumina Novaseq platform to generate 150 bp paired-end reads.

The characteristics of RNA-seq data were determined using principal component analysis (PCA) plots. PCA was completed using the ggfortify package in R (https://github.com/sinhrks/ggfortify). Samples in each group were located far from the other group samples in the gene expression PCA plot, which suggested that the RNA-seq quality was good. However, one sample of group 3 was located far from the other samples of group 3 in the gene expression PCA plot, for which this study excludes it (Figure S1A). The result of heat map and boxplot also showed that the data of this study were of good quality (Figure S1B-C).

### Differential expression analysis

The differentially expressed genes (DEGs), miRNAs (DEMs) and lncRNAs (DELs) were identified using the LIMMA package of R software (version 3.22.3; http://www.bioconductor.org/packages/release/bioc/html/edgeR.html).

### CeRNA regulatory network construction

We used the DIANA-LncBase v2 database (http://carolina.imis.athena-innovation.gr/diana_tools/web/index.php?r=lncbasev2%2Findex-experimental) to analyze the interactions between DELs and DEMs, and only retained DEL-DEM interactions with opposite electron expression trends. Also, we used StarBase to predict the target genes of DEMs (version 2.0; http://starbase.sysu.edu.cn/index.php). Subsequently, the set of DEM-DEG interactions was obtained by overlapping the target genes of DEMs with DEGs and retaining the negative interaction pairs between DEMs and DEGs. Finally, the DEL-DEM-DEG ceRNA axis was obtained by integrating the DEL-DEM and DEM-DEG interaction pairs, which were used to construct the ceRNA network and visualized using Cytoscape software (version 3.6.1; www.cytoscape.org/). Finally, we used the R program package called clusterprofiler to predict the Kyoto Encyclopedia of Genes and Genomes (KEGG) pathways and Gene Ontology (GO) terms for DEGs in the ceRNA network. Pathways or terms with a *p* value < 0.05 were considered statistically significant.

### Single cell analysis

In the Gene Expression Omnibus (GEO) database (http://www.ncbi.nlm.nih.gov/geo/), we selected GSE158055 for single-cell analysis and downloaded the sequencing data of the epithelial cells. Single-cell analysis was performed using the ‘Seurat’ package in the R (4.2.1). After standardizing the downloaded data, genes with large coefficient of variation between cells were extracted. Then, PCA was performed, t-distributed random neighborhood embedding (t-SNE) was used, and then marker genes were further found and differential analysis was performed to achieve the purpose of validation.

### Statistical analysis

We completed statistical analyses by using SPSS 23.0 (IBM, USA) and GraphPad Prism 9 (GraphPad, USA) and expressed the data results as the mean SD of three independent experiments. Comparisons between two groups were performed using Student’s t-test, and multiple comparisons were assessed by performing one-way ANOVA. Statistical significance was set at *p* value < 0.05. All experiments were performed in triplicate.

## Results

### N protein inhibits the induction of IFN-β in COVID-19

**Fig. 1:**
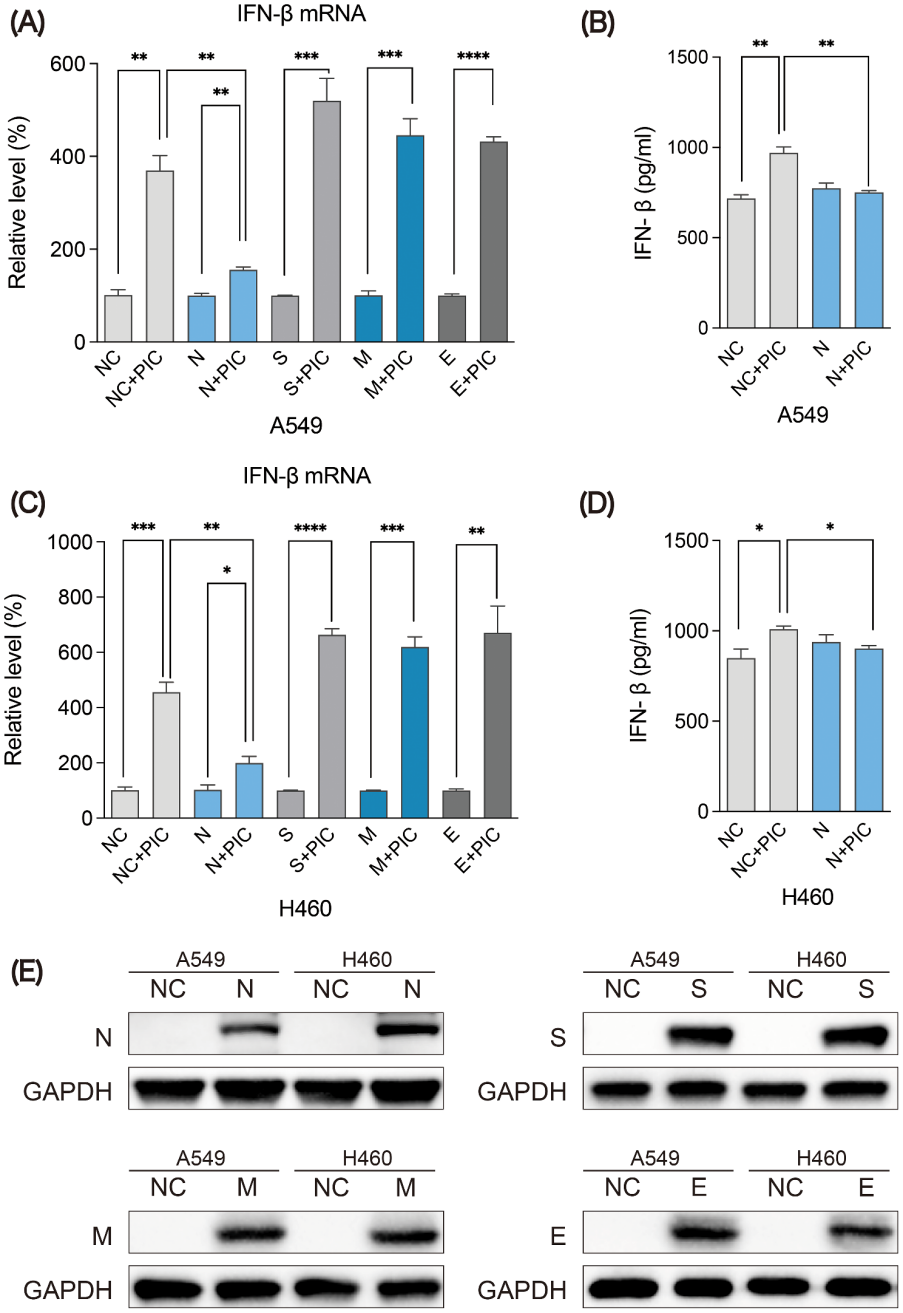
Effect of SARS-CoV-2 encoded proteins on the induction of IFN-β. Data are presented as means ± SD of three independent experiments. The mRNA expression of IFN-β in A549 cells (**A**) and H460 cells (**C**) with SARS-CoV-2 encoded proteins transfection and PIC treatment. ELISA was used to assess the induction level of IFN-β in A549 cells (**B**) and H460 cells (**D**) after treatment with N protein and PIC. (**E**) WB was used to verify the expression level of SARS-CoV-2 encoded proteins in A549 and H460 cells transfected with N, S, M and E expression plasmids. *p < 0.05, **p < 0.01, ***p < 0.001, ****p < 0.0001

To investigate the effect of SARS-CoV-2 structural proteins on the host innate immunity, A549 was independently transfected with N, S, M and E expression plasmids, then PIC was added and NC plasmid was used as the control. The results showed that only N protein inhibited the induction of *IFN-β* mRNA by PIC treatment (Fig. 1A). Compared with NC group, N protein also significantly inhibited the stimulation of IFN-β protein (Fig. 1B). The H460 cells obtained the consistent results (Fig. 1C-D). And the expression levels of the four encoded proteins were validated in A549 and H460 cells through WB (Fig. 1E). Altogether, our results indicate that N protein can inhibit the induction of IFN-β during SARS-CoV-2 invasion.

### Identification of genes related to N protein with PIC

**Fig. 2:**
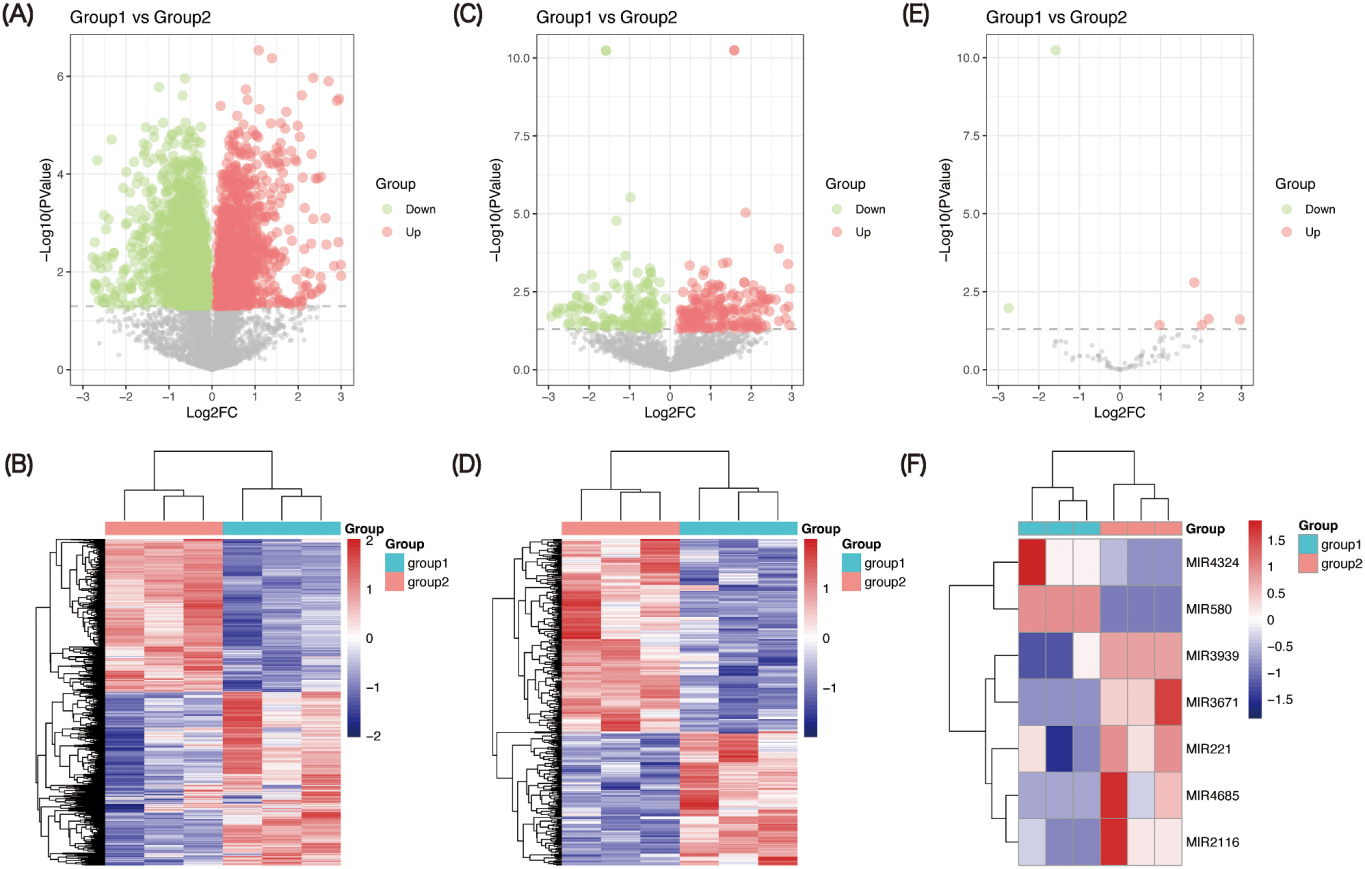
Expression patterns of mRNAs, lncRNAs and miRNAs in NC (group 1) and NC with PIC treatment (group 2). Volcano plot showing the regulation of differentially expressed mRNAs (**A**), lncRNAs (**C**) and miRNAs (**E**) between two groups. Green symbol shows down-regulation differentially expressed genes; red symbol shows up-regulation differentially expressed genes; gray symbol indicates nonsense genes that have been filtered out. Heat map demonstrating the expression of mRNAs (**B**), lncRNAs (**D**) and miRNAs (**F**) in the different samples. Red indicates a higher relative expression, while blue indicates a lower relative expression

A total of 11,136 mRNAs, 6,768 lncRNAs and 884 miRNAs were identified from the RNA-seq data. To analyze the inducible genes of PIC, we filtered for differential genes that met stringent *p* value (< 0.05) cutoffs, a total of 3,246 protein-coding genes (1737 down-regulated and 1509 up-regulated) (Fig. 2A-B), 339 lncRNAs (139 down-regulated and 200 up-regulated) (Fig. 2C-D) and 7 miRNAs (2 down-regulated and 5 up-regulated) (Fig. 2E-F) were identified from the gene expression data of NC (group 1) and NC with PIC treatment (group 2).

**Fig. 3:**
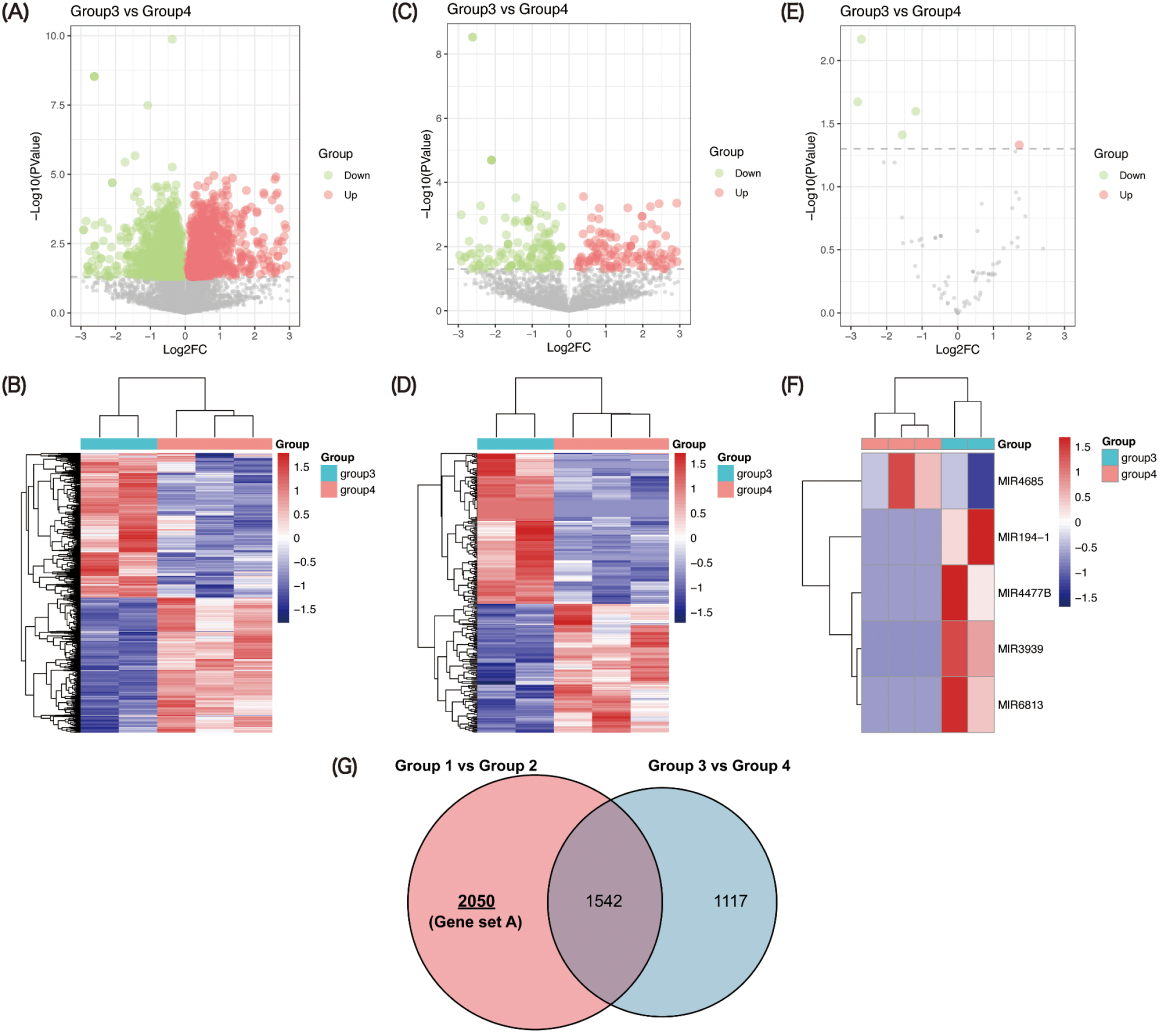
Expression patterns of mRNAs, lncRNAs and miRNAs in N protein (group 3) and N protein with PIC (group 4). Volcano plot showing the regulation of differentially expressed mRNAs (**A**), lncRNAs (**C**) and miRNAs (**E**) between two groups. Green symbol shows down-regulation differentially expressed genes; red symbol shows up-regulation differentially expressed genes; gray symbol indicates nonsense genes that have been filtered out. Heat map demonstrating the expression of mRNAs (**B**), lncRNAs (**D**) and miRNAs (**F**) in the different samples. Red indicates a higher relative expression, while blue indicates a lower relative expression. (**G**) Venn diagram of overlapping RNAs between differentially expressed RNAs in group 1 vs. group 2 and group 3 vs. group 4

Then we analyzed the differential genes of N protein with PIC. Using *p* value < 0.05 as the cutoffs, a total of 2378 protein-coding genes (1241 down-regulated and 1137 up-regulated) (Fig. 3A-B), 276 lncRNAs (149 down-regulated and 127 up-regulated) (Fig. 3C-D) and 5 miRNAs (4 down-regulated and 1 up-regulated) (Fig. 3E-F) were identified from the gene expression data of N protein (group 3) and N protein with PIC (group 4). One of the identification strategies of genes related to N protein was shown in Fig. 3G, and the candidate genes were obtained in red area of the venn diagram (gene set A).

### Differential gene analysis of N protein inhibiting IFNs induction

**Fig. 4:**
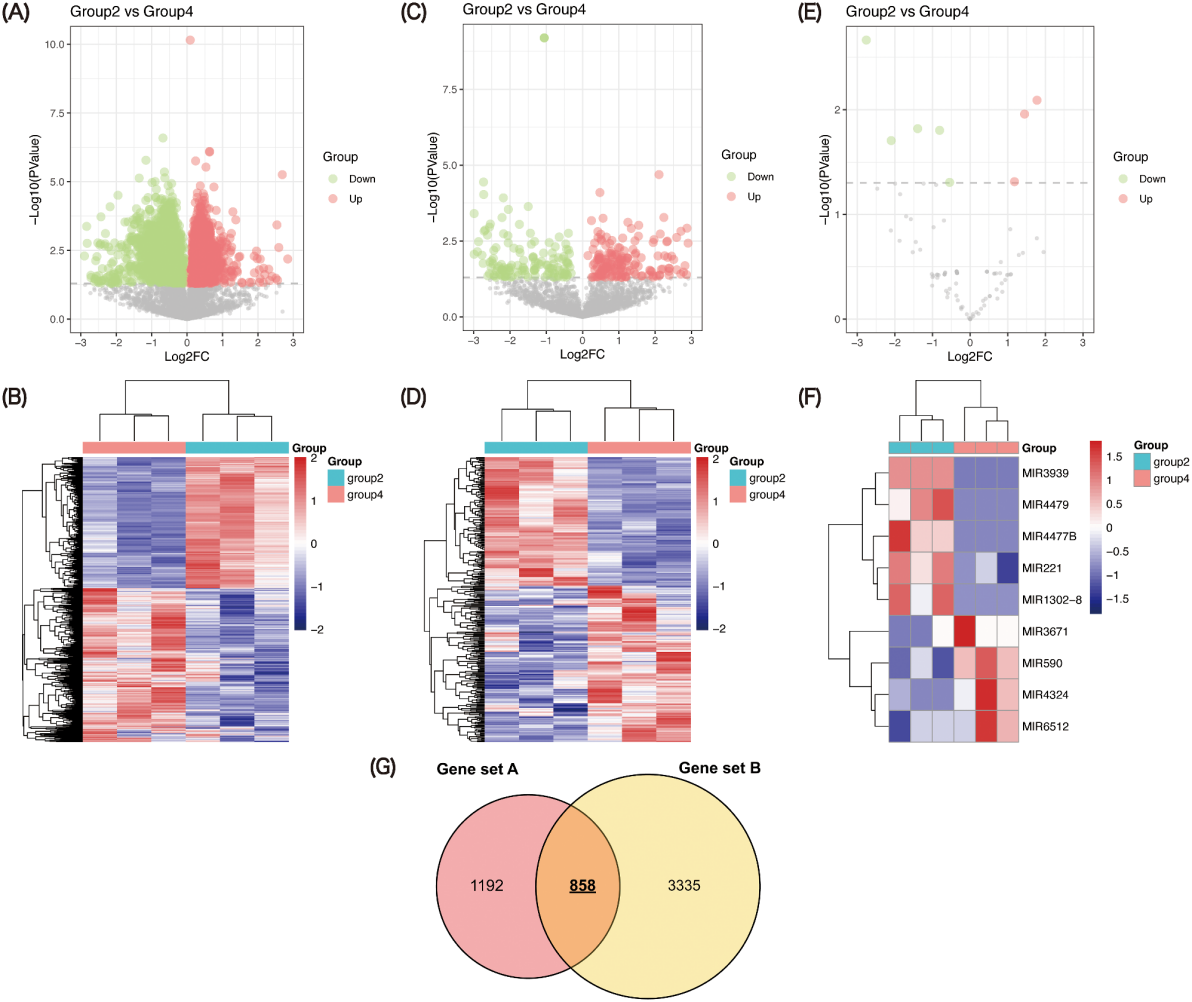
Expression pattern of mRNAs, lncRNAs and miRNAs in NC with PIC treatment (group 2) and N protein with PIC (group 4). Volcano plot showing the regulation of differentially expressed mRNAs (**A**), lncRNAs (**C**) and miRNAs (**E**) between two groups. Green symbol shows down-regulation differentially expressed genes; red symbol shows up-regulation differentially expressed genes; gray symbol indicates nonsense genes that have been filtered out. Heat map demonstrating the expression of mRNAs (**B**), lncRNAs (**D**) and miRNAs (**F**) in the different samples. Red indicates a higher relative expression, while blue indicates a lower relative expression. (**G**) Venn diagram of overlapping RNAs between differentially expressed RNAs in gene set A and gene set B

To obtain more accurate SARS-CoV-2 N protein-associated genes, the second differential gene analysis strategy was designed. As results, a total of 3874 protein-coding genes (1779 down-regulated and 2095 up-regulated) (Fig. 4A-B), 310 lncRNAs (140 down-regulated and 170 up-regulated) (Fig. 4C-D) and 9 miRNAs (5 down-regulated and 4 up-regulated) (Fig. 4E-F) were identified from the gene expression data of group 2 and group 4 (gene set B). Eventually, we overlapped the common genes in gene set A and gene set B, the 858 common genes were thought as the really genes related to N protein inhibiting IFNs induction (Fig. 4G).

**Fig. 5:**
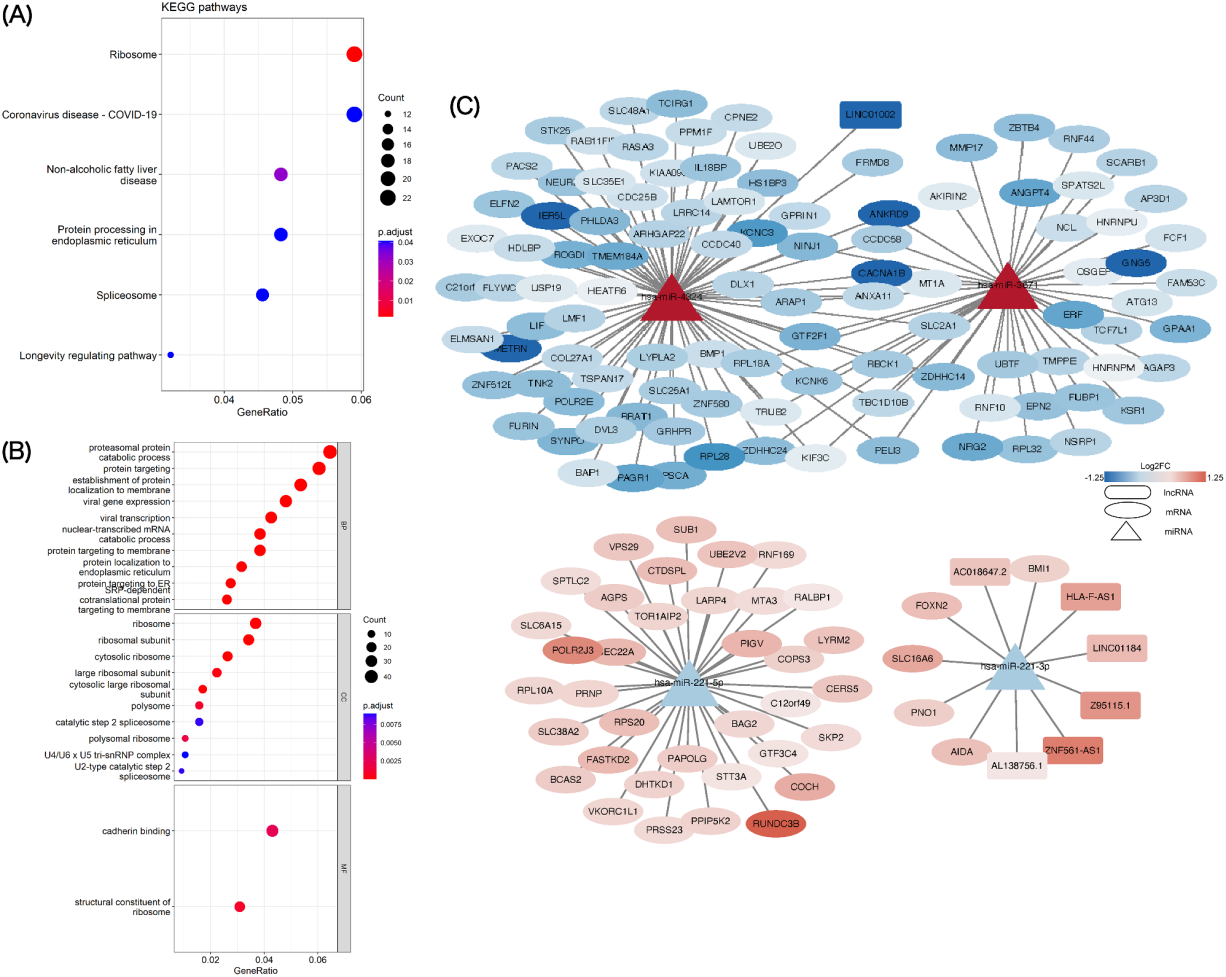
GO and KEGG functional analyses and interaction network of lncRNA‑miRNA‑mRNA ceRNA. KEGG pathway enrichment (**A**) and GO analysis (**B**) of DEGs. (**C**) Interactive lncRNA-miRNA-mRNA ceRNA network. Square nodes represent lncRNAs; triangle nodes represent miRNAs; oval nodes represent mRNAs

KEGG and GO pathway enrichment analyses were performed for the DEGs to predict their functions. KEGG results revealed that 6 pathways were also enriched, including hsa03010: Ribosome, hsa04932: Non-alcoholic fatty liver disease, hsa03040: Spliceosome, hsa04211: Longevity regulating pathway, hsa04141: Protein processing in endoplasmic reticulum, hsa05171: Coronavirus disease-COVID-19 (Fig. 5A).

The most enriched GO analysis term for the biological processes was viral gene expression (GO: 0019080) and viral transcription (GO: 0019083); for the cellular component and the molecular function was related to ribosome synthesis, including cytosolic ribosome (GO: 0022626), ribosomal subunit (GO: 0044391), structural constituent of ribosome (GO: 0003735) (Fig. 5B).

### CeRNA network associated with N protein

We predicted 162 DEG-DEM interaction pairs (with opposite expression trends) between 144 DEGs (103 down-regulated and 41 up-regulated) and 4 DEMs (2 down-regulated and 2 up-regulated) using the TargetScan database. Subsequently, we predicted the lncRNAs of the above mentioned 4 DEMs by DIANA-LncBase v2 database. After removing DEMs and DELs with consistent expression trends, we finally retained the DEL-DEM interaction pairs between 7 DELs (1 down-regulated and 6 up-regulated) and 2 DEMs (1 down-regulated and 1 up-regulated), and constructed a DEL-DEM-DEG ceRNA network based on this. The network consisted of 155 nodes, including 7 DELs (1 down-regulated and 6 up-regulated), 4 DEMs (2 down-regulated and 2 up-regulated), and 144 DEGs (103 down-regulated and 41 up-regulated) and we visualized the SARS-CoV-2 N protein-associated gene interactome through a network shown in Fig. 5C.

### Validation of gene expression pattern of genes related to N protein in single cell level

**Fig. 6:**
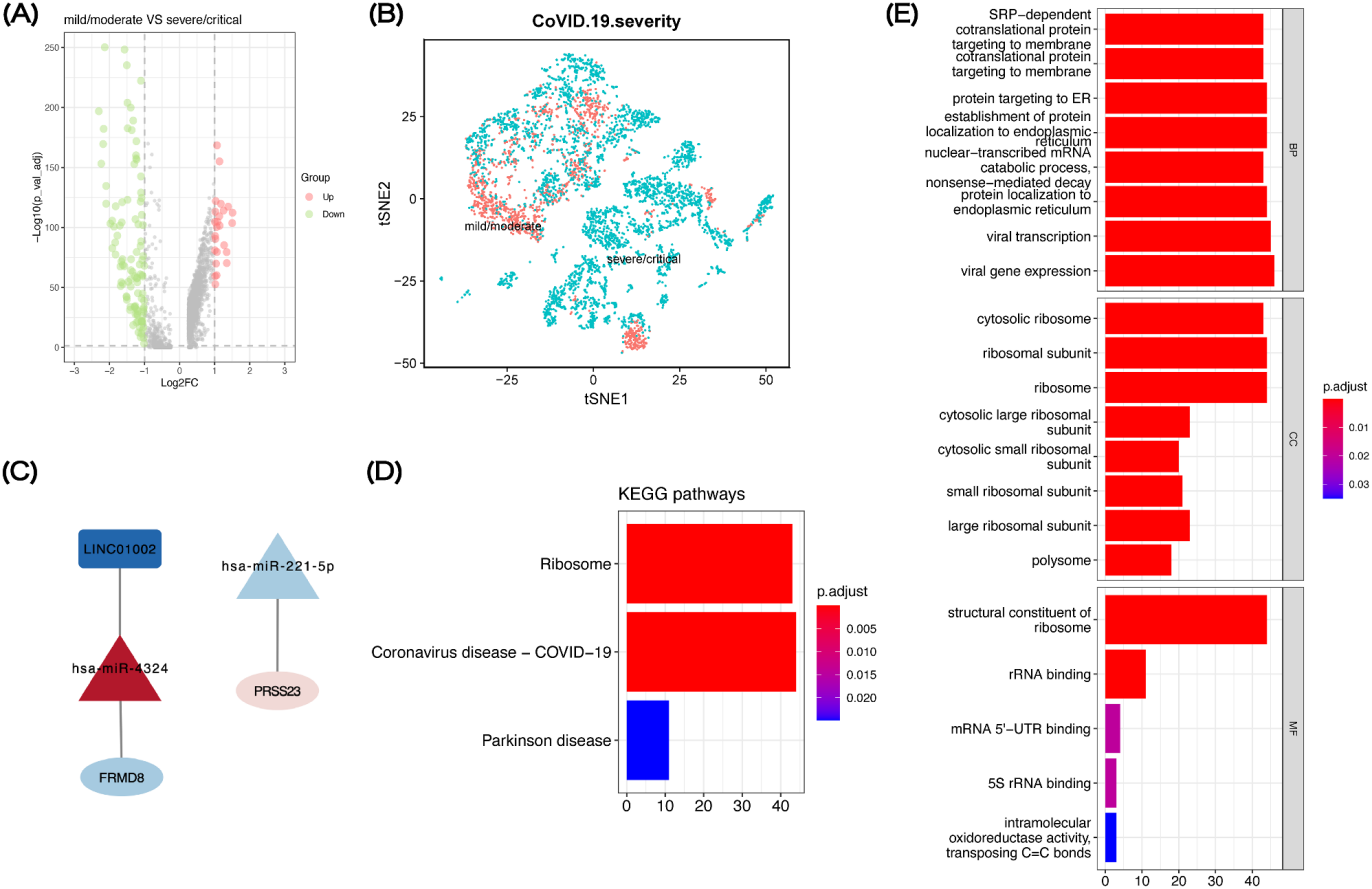
Validation and core gene analysis at the single cell level. (**A**) Volcano plots show differentially expressed genes between the mild/moderate and the severe/critical in single cell dataset (GSE158055). Green symbol shows down-regulation differentially expressed genes; red symbol shows up-regulation differentially expressed genes; gray symbol indicates nonsense genes that have been filtered out. (**B**) t-SNE clustering of COVID-19. (**C**) Two candidate ceRNA axes obtained from all analysis data. KEGG pathways (**D**) and GO functional analyses (**E**) of DEGs between the mild/moderate and the severe/critical

In order to validate the result of the RNA-seq data, we downloaded the single cell dataset of GSE158055. Total 5652 epithelial cells were downloaded, which had been quality-controlled for further analysis (Fig. 6A). Following gene expression normalization, we conducted dimensionality reduction and clustering using principal component analysis and t-SNE, respectively. In this study, we identified 129 differentially expressed genes (|log2FC| > 1 and adjusted *p*-value < 0.05) between epithelial cells from mild/moderate patients and epithelial cells from severe/critical patients (Fig. 6B). There were 2 DEGs (FRMD8, PRSS23) in both RNA-seq data above and single cell data.

### CeRNA network of core gene related to N protein

Based on the ceRNA network containing FRMD8 and PRSS23, we discovered the LINC01002-miR-4324-FRMD8 and miR-221-5p-PRSS23 ceRNA axes (Fig. 6C) that may function as candidate axes of induction of IFN-β by N proteins. KEGG analysis indicated significant enrichment for Coronavirus disease-COVID-19 and Ribosome (Fig. 6D). And GO analysis of epithelial cells indicated significant enrichment for viral transcription, viral gene expression, and ribosome (Fig. 6E).

**Fig. 7:**
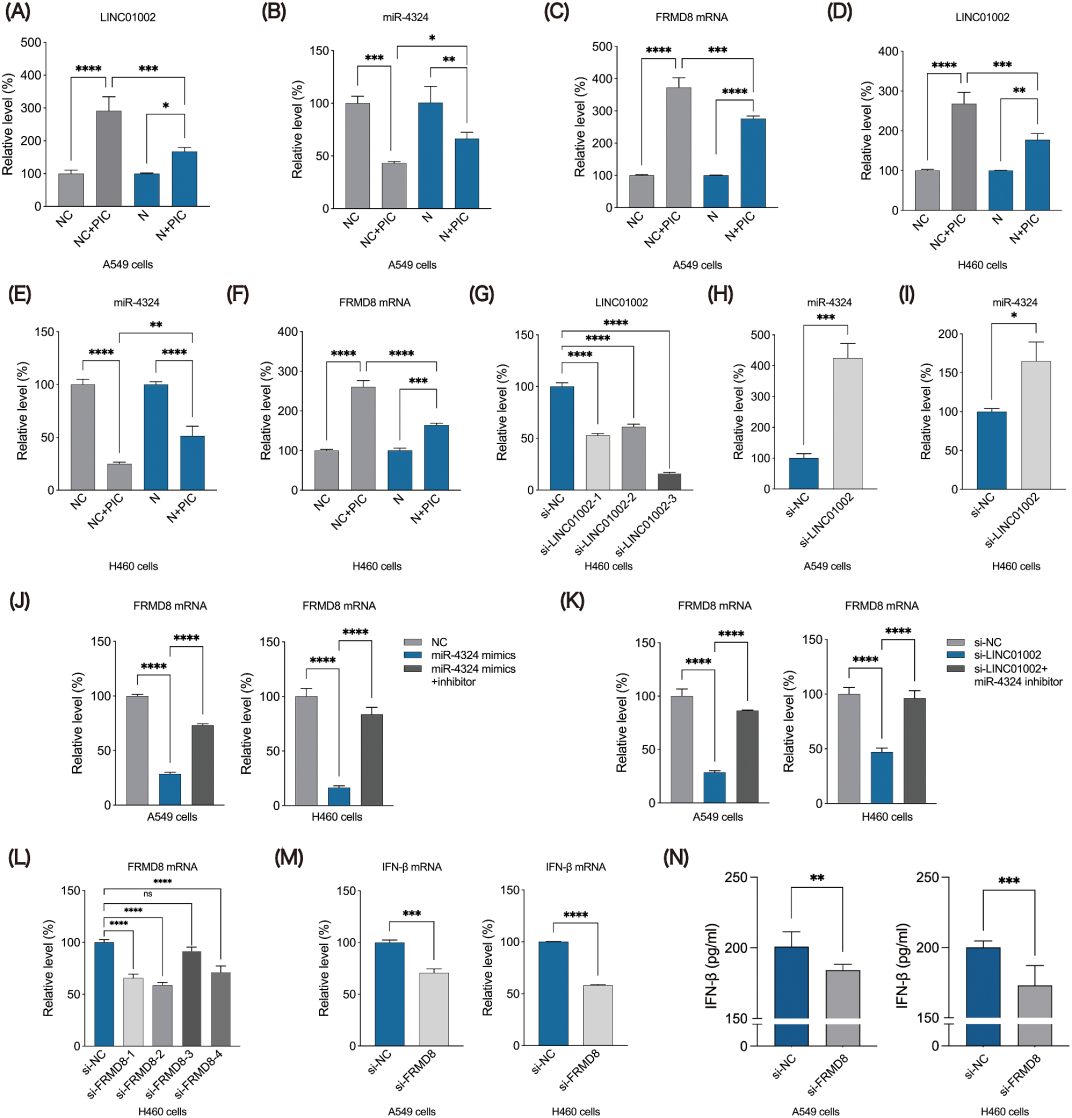
LINC01002 via sponging miR-4324 to regulate FRMD8 in the process of N protein with PIC treatment. Expression levels of LINC01002 (**A**), miR-4324 (**B**) and FRMD8 (**C**) in A549 cells transfected with NC with PIC treatment and N protein with PIC. (**D**-**F**) Expression levels of LINC01002, miR-4324 and FRMD8 in H460 cells, grouped as above. (**G**) Screening the effective siRNA for LINC01002 knockdown. The role of silencing LINC01002 on the expression of miR-4324 in A549 cells (**H**) and H460 cells (**I**). (**J**) The expression of FRMD8 mRNA in A549 cells (left) and H460 cells (right) after up-regulating miR-4324 or transfecting miR-4324 mimics and inhibitors meanwhile. (**K**) The expression of FRMD8 mRNA in A549 cells (left) and H460 cells (right) after down-regulating LINC01002 or transfecting si-LINC01002 and miR-4324 inhibitors meanwhile. (**L**) Screening the effective siRNA for the knockdown of FRMD8. The levels of IFN-β mRNA (**M**) and protein (**N**) expression in A549 cells and H460 cells after FRMD8 knockdown. *p < 0.05, **p < 0.01, ***p < 0.001, ****p < 0.0001

### N protein suppresses IFN-β induction via LINC01002-miR-4324-FRMD8 axis

PRSS23 has been well established on SARS-CoV-2 in a recent research [29], but FRMD8 still needs to be explored in this related field. Therefore, we further explored the induction of IFNs by N protein and LINC01002-miR-4324-FRMD8 axis. It was found that PIC can induce the up-regulation of LINC01002 and FRMD8 mRNA, and the facilitative effect can be partially inhibited by SARS-CoV-2 N protein in A549 cells (Fig. 7A-C). In contrast, miR-4324 showed a completely opposite trend, and SARS-CoV-2 N protein could reverse the inhibitory effect of PIC on miR-4324 level (Fig. 7B). Similar to the results above, H460 cells also demonstrated the regulation of LINC01002-miR-4324-FRMD8 axis by SARS-CoV-2 N protein (Fig. 7D-F). To verify the relationship among three key genes, we constructed three different siRNAs of LINC01002 and selected the si-LINC01002-3 with the best knockdown effect (Fig. 7G). Moreover, the results showed that the level of miR-4324 increased significantly after knockdown of LINC01002 (Fig. 7H-I). The elevated and decreased expression of miR-4324 also significantly affected the production of FRMD8 (Fig. 7J). Furthermore, suppressing miR-4324 could counteract the effect of down-regulating LINC01002 on FRMD8 mRNA expression (Fig. 7K). In addition, we designed four different siRNAs targeting FRMD8 to screen the most effective one (si-FRMD8-2) for FRMD8 knockdown for subsequent experiments (Fig. 7L). We discovered that after FRMD8 knockdown, the mRNA and protein expression levels of IFN-β were significantly decreased (Fig. 7M-N). All these results have confirmed that SARS-CoV-2 suppresses IFN-β induction via N protein-dependent LINC01002-miR-4324-FRMD8 axis.

**Fig. 8:**
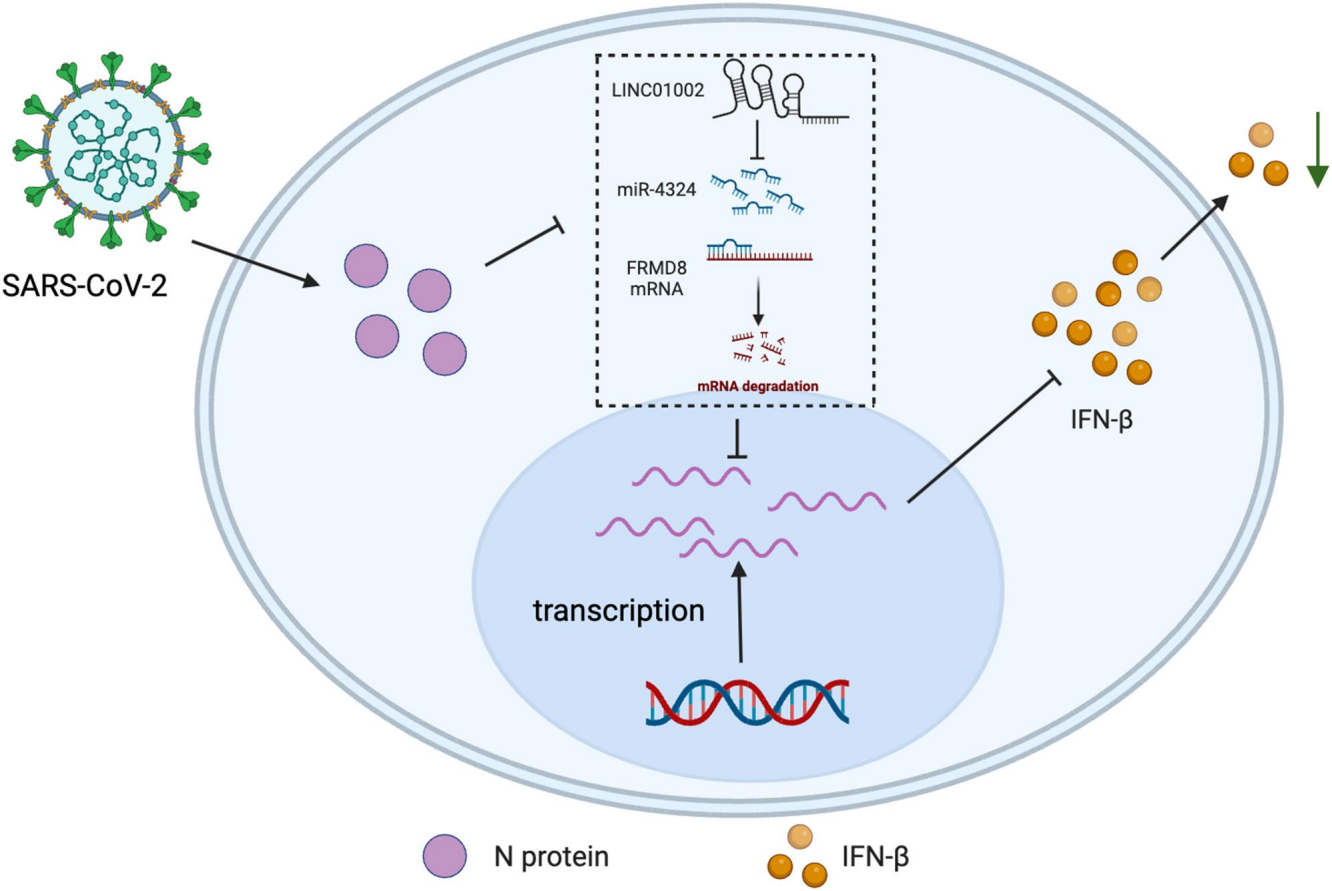
Schematic depiction of the regulatory role of SARS-CoV-2 N protein against IFN-β signaling

## Discussion

SARS-CoV-2 is a novel coronavirus that was discovered in 2019 and is phylogenetically related to SARS-CoV-1 and bat-associated SARS-CoV [30, 31]. Coronaviruses such as SARS-CoV-1 may impair IFNs production by evading recognition by pattern recognition receptors (PRRs), interfering with RIG-I or TLR signaling, and inhibiting IRF3 activation [32–34]. Previous study demonstrated that different arms of the type I IFN induction pathway can be blocked by the expression of SARS-CoV-1 ORF3b, ORF6, and N protein in vitro [35]. Additionally, SARS-CoV-1 may disrupt the signaling cascade downstream of IFNs production by inhibiting STAT1 nuclear translocation [36]. Similar to SARS-CoV-1, SARS-CoV-2 also inhibits IFNs induction and signaling by various ways. In comparison, SARS-CoV-2 induces more reduction in pro-inflammatory cytokines and chemokines and more potent inhibition of type I/III and type II IFN [37]. Type I/ III IFN responses are the primary mechanisms of innate antiviral immunity in infection clearance, while type I IFN induction deficiency could be a distinguishing feature of severe COVID-19 [13, 38]. A number of SARS-CoV-2 proteins were reported to antagonize type I IFN responses [18]. In this research, PIC acts as a dsRNA mimic to induce the production of type I IFN, including IFN-β [28]. The PIC we used is considered to be low molecular mass-PIC (LMM-PIC). A study showed that high molecular mass-PIC (HMM-PIC) is more effective in inducing IFNs than LMM-PIC [39]. Therefore, the replacement from LMM-PIC to HMM-PIC is an issue for us to further explore in future experimental validations.

Research has demonstrated that SARS-CoV-2 proteins can inhibit various steps in the production and response of type I IFN [40]. During SARS-CoV-2 infection, viral RNA is recognized by RIG-I and melanoma-differentiation-associated gene 5 (MDA5) and activates the downstream MAVS/TBK1/IRF3 axis, initiating the expression of type I IFN [41]. As a poor inducer of type I IFN, SARS-CoV-2 inhibits not only core cellular functions, such as transcription and translation, but also various molecules involved in type I IFN production specifically. About half the known SARS-CoV-2 proteins target human proteins in the type I IFN induction pathways [18]. NSP1 has been proven to inhibit type I IFN production and response by inhibiting the association of IRF3 with the IFN-β promoter, the protein translation and preventing mRNA from entering ribosome [42]. NSP6 and NSP13 antagonize IFN-β production by targeting IRF3 or another component upstream of IRF3 (between TBK1/IKKε and IRF3), while ORF6 inhibits IFN-β production by binding to karyopherin subunit alpha 2 (KPNA2) to block IRF3 nuclear translocation [18]. In addition, the formation of NSP14-NSP10 complex enhances translation inhibition executed by NSP14, abolishing the type I IFN-dependent induction of interferon-stimulated genes (ISGs) and inhibiting the production of antiviral proteins [43]. NSP1, NSP5, NSP6, NSP15, ORF6 and ORF7b strongly (> 10-fold) blocked MAVS-induced IFN-β production, inducing a collapse in IFN-β mRNA levels and corresponding with suppressed IFN-β protein secretion [19]. In addition to non-structural and accessory proteins, structural proteins have been proven to be crucial type I IFN antagonists. Here, in order to enhance the reliability of the study, we used two most common cell lines of SARS-CoV-2 to conduct the experiments. Through screening various structural proteins, we found that the one that plays a pivotal role in the inhibition of IFN-β induction by SARS-CoV-2 is N protein. Our results showed that the N protein can inhibit the induction of IFN-β in both A549 and H460 cells. However, the suppression level of IFN-β protein in H460 cells was slightly lower than in A549 cells. Considering the same treatment conditions and operational procedures during the experiments, and the transfection efficiency of the cells was consistent, the inconsistency in the suppression ability of N protein on IFN-β induction between the two cell lines may be due to the differences in cell lines. These results of our study were also similar to the previous study of SARS-CoV-2 [44]. Moreover, further studies on the innate immunosuppression by SARS-CoV-2 N protein will elucidate the viral pathogenesis of COVID-19 disease.

Studies that focus on understanding the inhibitory effects of nucleocapsid proteins on the innate immune response in various viruses, including hepatitis C virus (HCV), influenza A virus (IAV) and infectious hematopoietic necrosis virus, have made considerable progress [45–47]. During the invasion of SARS-CoV-2 into host cells, N protein binds to the DExD/H domain of RIG-I inhibiting the activation of an IFN-β promoter reporter, endogenous IFN-β mRNA expression, and IRF3 activation in response to Sendai virus and transfection with PIC [48]. Our findings indicate that the low-dose N protein can suppress IFN-β production, aligning with the studies by Thorne LG and Chen K [25, 26]. Whereas, Zhao Y et al. found that the high-dose N protein promoted type I IFN signaling and inflammatory cytokines [27]. Therefore, varying doses of SARS-CoV-2 N protein may play a dual-role in regulating IFN-β expression. Moreover, the role of the high-dose N protein may be a direction of our future research. Besides, the specific mechanisms by which N proteins lead to reduced IFN-β induction are less comprehensive. Our research targeting SARS-CoV-2 N protein in immune escape and IFN-β induction suppression will be an essential addition.

Here, our study reveals that SARS-CoV-2 N protein inhibits the induction of IFN-β, playing a pivotal role in severe inflammatory responses and immunopathological changes of COVID-19. LncRNAs and miRNAs are involved in the pathogenesis of SARS-CoV-2 and the host antiviral immune defense mechanism, and lncRNAs can be used as sponges of miRNAs to reduce miRNA degradation of their target mRNA [49]. Studies have shown that lncRNA Gm26917 can reduce the degradation of RIG-I by sponging miR-124-3p, thus inferring that network interactions of this ceRNA have the potential to increase the chance of SARS-CoV-2 replication [50]. MiR-4324 has been shown in several studies to inhibit tumor proliferation, migration, and epithelial-mesenchymal transition in a variety of tumor cells, including breast, bladder, ovarian cancers and esophageal squamous-cell carcinoma [51, 52]. Previous study has identified that FRMD8 is essential for the control of the stability of inactive rhomboid protein 2 (iRhom2) and a disintegrin metalloprotease 17 (ADAM17) on the plasma membrane. Ablation of FRMD8 triggers the mis-sorting of iRhom2 and ADAM17 to lysosomes, where they are degraded [53]. Consistent with this, loss of FRMD8 results in a dramatic reduction in ADAM17 activity and tumor necrosis factor (TNF) secretion, revealing FRMD8 as a key physiological regulator of TNF release. On the other side, TNF triggers a type I IFN response and an early and robust type I IFN response is required as the very first line of defense to suppress viral replication and spread [54, 55]. Besides, FRMD8 negatively regulates the WNT/β-catenin (CTNNB1) signaling, thereby preventing the recruitment of the signal transduction protein axin [56]. Previous research shows that inhibition of the kinase glycogen synthase kinase 3 (GSK-3) could enhance the constitutive level of IFN-β expression, provided that it leads to the interaction of CTNNB1 with the IFN-β promoter [57]. WNT modulates the secretion of IFN-β through the CTNNB1 pathway during viral infection, while knockdown of CTNNB1 notably increased secretion of IFN-β protein [58]. Collectively, these studies indicate that the WNT/CTNNB1 signaling pathway is correlated with the expression of IFN-β, whereas FRMD8 may regulate IFN-β expression via the WNT/CTNNB1 signaling pathway. Our results showed that knockdown of FRMD8 significantly inhibits the induction of IFN-β, which is consistent with the above finding. Besides, further research is required to elucidate the specific mechanism. In this article, through two differential analysis strategies based on RNA-seq data, N protein-related genes were identified for the construction of ceRNA network, revealing the process of gene regulation when SARS-CoV-2 virus infects cells. By combing single cell sequencing, we have verified that the ceRNA axis of LINC01002-miR-4324-FRMD8 may play an important regulatory role in SARS-CoV-2 infected cells for the first time (Fig. 8). Moreover, the synergistic effect of this ceRNA axis has brought new breakthroughs in the study of COVID-19.

## Conclusion

Currently, there are no specific and effective drugs available for the clinical treatment of COVID-19. And this lncRNA-miRNA-mRNA axis could influence the IFN-β induction and subsequently virus replication and propagation. Thus, it provides a relevant target for early intervention therapy and drug development of COVID-19. Given that our study was conducted in vitro, further investigations using an animal model or clinical studies are highly valuable for understanding the human immune response during COVID-19 infection, which could be significant for the diagnosis and treatment.

### Electronic supplementary material

Below is the link to the electronic supplementary material.

Supplementary Material 1

Supplementary Material 2

## Data Availability

No datasets were generated or analysed during the current study.

## Abbreviations

COVID-19: Coronavirus disease 2019
SARS-CoV-2: Syndrome coronavirus-2
IFN-β: Interferon-beta
RNA-Seq: RNA-sequencing
ceRNA: Competitive endogenous RNA
FRMD8: FERM domain-containing protein 8
PIC: Polyinosinic-polycytidylic acid
MERS-CoV: Middle East respiratory syndrome coronavirus
ARDS: Acute respiratory distress syndrome
MODS: Multiple organ dysfunction syndrome
CSSE: Center for Systems Science and Engineering
gRNA: Genomic RNA
sgRNA: Subgenomic rnas
IFNs: Interferons
ORF: Open reading frame
NSPs: Nonstructural proteins
MAVS: Mitochondrial antiviral signaling proteins
TRAF3: TNF receptor-associated factor 3
TBK1: TANK-binding kinase 1
IRF3: Mitochondrial antiviral signaling proteins
RIG: Retinoic acid-inducible gene
DMEM: Dulbecco’s modified eagle medium
FBS: Fetal bovine serum
PCA: Principal component analysis
DEGs: Differentially expressed genes
DEMs: Differentially expressed mirnas
DELs: Differentially expressed lncrnas
KEGG: Kyoto Encyclopedia of Genes and Genomes
GO: Gene ontology
GEO: Gene expression omnibus
t-SNE: T-distributed random neighborhood embedding
PRR: Pattern recognition receptors
MDA5: Melanoma-differentiation-associated gene 5
KPNA2: Karyopherin subunit alpha 2
ISGs: Interferon-stimulated genes
HCV: Hepatitis C virus
IAV: Influenza A virus
iRhom2: Inactive rhomboid protein 2
ADAM17: A disintegrin metalloprotease 17
TNF: Tumor necrosis factor
