# Supplementary material for: LINC01002 functions as a ceRNA to regulate FRMD8 by sponging miR-4324 for the development of COVID-19
Source: Virol J. 2024 May 11;21:109. doi: 10.1186/s12985-024-02382-2 (PMC11088083; doi:10.1186/s12985-024-02382-2)
Supplement: Supplementary file 1 — Supplementary Material 1 [file 12985_2024_2382_MOESM1_ESM.docx]

**TABLE S1** RNA oligo used for cell transfection

| RNA oligo Type |  | Sequence (5'-3') |
| --- | --- | --- |
| si-NC | S | UUCUCCGAACGUGUCACGUdTd |
|  | AS | ACGUGACACGUUCGGAGAAdTdT |
| si-LINC01002-1 | S | AGACCAAGCUCAUGACUCACATT |
|  | AS | UGUGAGUCAUGAGCUUGGUCUTT |
| si-LINC01002-2 | S | GCAUCUACCUCACUGUGGACCTT |
|  | AS | GGUCCACAGUGAGGUAGAUGCTT |
| si-LINC01002-3 | S | GAUUCCUGCCUCUCAACAACCTT |
|  | AS | GGUUGUUGAGAGGCAGGAAUCTT |
| NC | S | UUCUCCGAACGUGUCACGUTT |
|  | AS | ACGUGACACGUUCGGAGAATT |
| miR-4324 mimics | S  AS | CCCUGAGACCCUAACCUUAA  AAGGUUAGGGUCUCAGGGUU |
| miR-4324 inhibitor | S  AS | UUAAGGUUAGGGUCUCAGGG |
| si-FRMD8-1 | S | GCUGGUAUACCUAGCGGAUTT |
|  | AS | AUCCGCUAGGUAUACCAGCTT |
| si-FRMD8-2 | S | CCGGCUGCAACAGUCUCAUTT |
|  | AS | AUGAGACUGUUGCAGCCGGTT |
| si-FRMD8-3 | S | GCCUUUGUGCCCACCUCAATT |
|  | AS | UUGAGGUGGGCACAAAGGCTT |
| si-FRMD8-4 | S | GGUGACCAGUCUCCGUCUUTT |
|  | AS | AAGACGGAGACUGGUCACCTT |

NC: negative control. S: sense. AS: antisense.

**TABLE S2** Primers used for RT-qPCR amplification

| Primer name |  | Sequence (5'-3') |
| --- | --- | --- |
| GAPDH[1] | F | GGACCTGACCTGCCGTCTAG |
|  | R | GTAGCCCAGGATGCCCTTGA |
| SARS-CoV-2-S | F | GTCCGTGATCCACAGACACTTGAG |
|  | R | TGCCCGCCGAGGAGAATTAGTC |
| SARS-CoV-2-N | F | CTCTTGCTTTGCTGCTGCTTGAC |
|  | R | AGGTGTGACTTCCATGCCAATGC |
| SARS-CoV-2-E | F | CGTTTCGGAAGAGACAGGTACG |
|  | R | AGACCAGAAGATCAGGAACTCTAGA |
| SARS-CoV-2-M | F | ACATTCTTCTCAACGTGCCACTCC |
|  | R | GCCAATCCTGTAGCGACTGTATGC |
| LINC01002 | F | TGTGAAGACTGGTGTGGGAAGG |
|  | R | ATGTAGAATTAGTGGGCGTGTCAAG |
| U6 | RT | TGGAACGCTTCACGAATTTGCG |
|  | F | GGAACGATACAGAGAAGATTAGC |
|  | R | TGGAACGCTTCACGAATTTGCG |
| miR-4324 | RT | GAAAGAAGGCGAGGAGCAGATCGAGGAAGAAGACGGAAGAATGTGCGTCTCGCCTTCTTTCTTAAGGTTGGAACGATACAGAGAAGATTAGC |
|  | F | GTAGGTTGTCCCTGAGACCCT |
|  | R | GAAAGAAGGCGAGGAGCAGATC  CTTCTTCCACGGTGAGGTTGAC |
| FRMD8 | F |  |
|  | R | GCACGCCTTCCAGACTGATG |
| IFN-β[2] | F | AAGGCCAAGGAGTACAGT |
|  | R | AGTTTCGGAGGTAACCTG |

F: forward primer. R: reverse primer. RT: reverse transcription primer.

1. Yu X, Chen Y, Cui L, Yang K, Wang X, Lei L, et al. CXCL8, CXCL9, CXCL10, and CXCL11 as biomarkers of liver injury caused by chronic hepatitis B. Front Microbiol. 2022;13:1052917.

2. Qian J, Zhai A, Kao W, Li Y, Song W, Fu Y, et al. Modulation of miR-122 on persistently Borna disease virus infected human oligodendroglial cells. Antiviral Res. 2010;87:249–56.
