# Supplementary material for: LINC01002 functions as a ceRNA to regulate FRMD8 by sponging miR-4324 for the development of COVID-19
Source: Virol J. 2024 May 11;21:109. doi: 10.1186/s12985-024-02382-2 (PMC11088083; doi:10.1186/s12985-024-02382-2)
Supplement: Supplementary file 2 — Supplementary Material 2 [file 12985_2024_2382_MOESM2_ESM.docx]

**
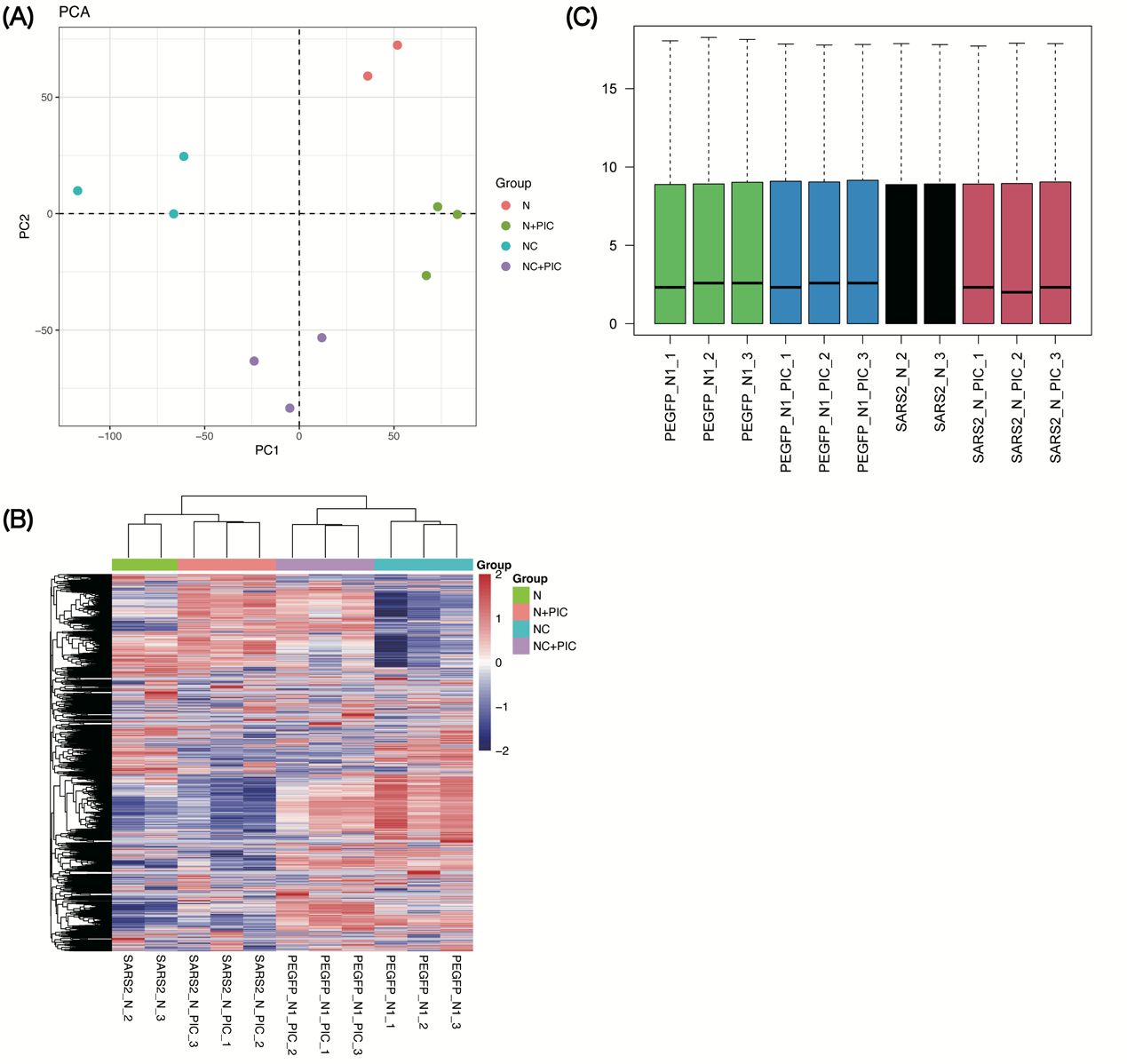
**

**FIGURE S1** Quality control of RNA-Seq data.

(A) PCA plot after quality control. (B) Heatmap showed the most variable 10000 genes between 4 types of groups in the RNA-Seq data. (C) Box plot after data standardization.
